# A Review of Machine Learning and QSAR/QSPR Predictions for Complexes of Organic Molecules with Cyclodextrins

**DOI:** 10.3390/molecules29133159

**Published:** 2024-07-02

**Authors:** Dariusz Boczar, Katarzyna Michalska

**Affiliations:** Department of Synthetic Drugs, National Medicines Institute, Chełmska 30/34, 00-725 Warsaw, Poland

**Keywords:** quantitative structure–activity relationship (QSAR), quantitative structure–property relationship (QSPR), machine learning, cyclodextrin, inclusion complex, Gibbs free energy (Δ*G*), stability constant

## Abstract

Cyclodextrins are macrocyclic rings composed of glucose residues. Due to their remarkable structural properties, they can form host–guest inclusion complexes, which is why they are frequently used in the pharmaceutical, cosmetic, and food industries, as well as in environmental and analytical chemistry. This review presents the reports from 2011 to 2023 on the quantitative structure–activity/property relationship (QSAR/QSPR) approach, which is primarily employed to predict the thermodynamic stability of inclusion complexes. This article extensively discusses the significant developments related to the size of available experimental data, the available sets of descriptors, and the machine learning (ML) algorithms used, such as support vector machines, random forests, artificial neural networks, and gradient boosting. As QSAR/QPR analysis only requires molecular structures of guests and experimental values of stability constants, this approach may be particularly useful for predicting these values for complexes with randomly substituted cyclodextrins, as well as for estimating their dependence on pH. This work proposes solutions on how to effectively use this knowledge, which is especially important for researchers who will deal with this topic in the future. This review also presents other applications of ML in relation to CD complexes, including the prediction of physicochemical properties of CD complexes, the development of analytical methods based on complexation with CDs, and the optimisation of experimental conditions for the preparation of the complexes.

## 1. Introduction

In chemistry, some compounds spark significant research interest due to their excellent physicochemical properties. Among them, cyclodextrins (CDs), discovered in 1891, are the subject of intense research worldwide. This is reflected in approximately 61,000 peer-reviewed scientific articles and approximately 32,000 patents published by the end of February 2023 [1].

**Figure 1:**
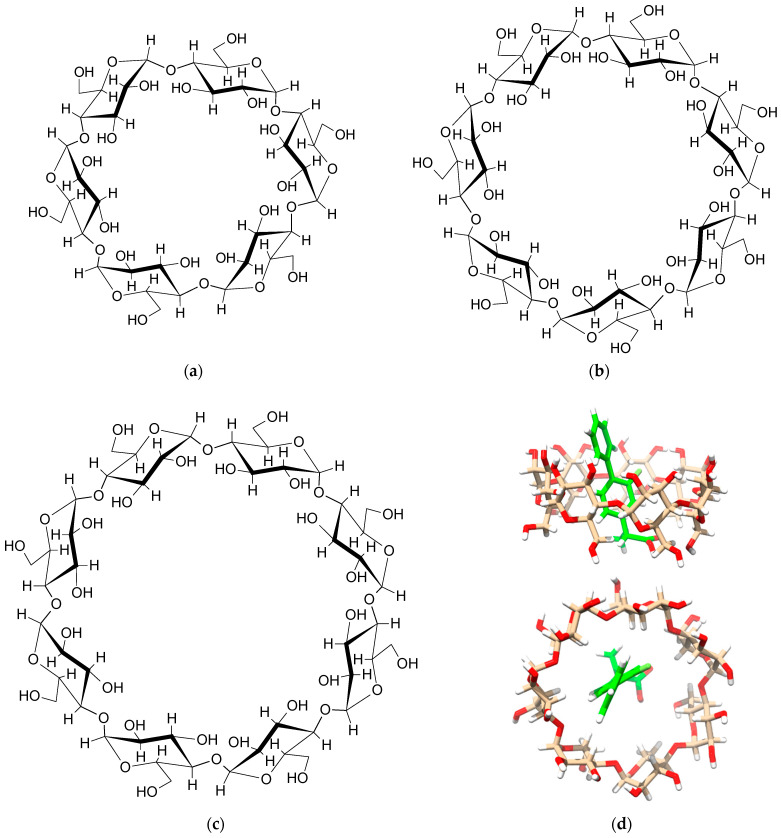
Structural formulas of (**a**) α-CD, (**b**) β-CD, (**c**) γ-CD, and (**d**) exemplary 3D structure of the β-CD–flurbiprofen inclusion complex based on crystallographic data [2]. The flurbiprofen molecule is shown in green.

**Figure 2:**
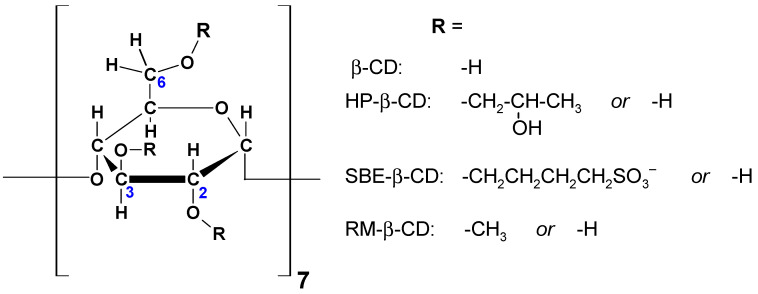
Structural formulas of HP-β-CD, SBE-β-CD, and RM-β-CD.

From a chemical point of view, CDs are macrocyclic rings composed of a specified number of glucose units: six for α-CD, seven for β-CD, and eight for γ-CD (Figure 1). Their three-dimensional (3D) structure bears resemblance to a torus or an even more truncated cone, featuring one narrow rim containing six, seven, or eight primary hydroxyl groups (-OH) linked to the C6 atoms of the glucopyranoside units. Additionally, a wide rim accommodates 12, 14, or 16 secondary hydroxyl groups attached to the C2 and C3 carbon atoms for α-, β-, and γ-CD, respectively. Refer to Figure 2 for detailed atom labelling. Interestingly, hydroxyl groups are located on the outer surface of CDs, which promotes intermolecular interactions with water and results in the favourable solubility of CDs in water. However, it should be mentioned that among the three native CDs, β-CD has the lowest solubility in water. This is because the β-CD molecule is an almost rigid body, except for the oxygen and hydrogen atoms in the hydroxymethyl group and the hydrogen atoms in the hydroxyl groups. On the other hand, the inner surface of CDs is hydrophobic, which means that it interacts more strongly with non-polar compounds that are sparingly soluble in water. This remarkable structural property of CDs enables the formation of inclusion (host–guest) complexes. In general, it is thermodynamically preferable for a non-polar, hydrophobic substance to enter and remain in the hydrophobic cavity of CDs, compared to being exposed to undesirable contact with water. Hence, the CD–guest inclusion complex dissolves easily in water due to the hydrophilic nature of the external surface of CDs.

It is worth mentioning that in addition to the three native CDs discussed so far (α-, β-, γ-CD), some derivatives of CDs have been synthesised with some of the -OH groups replaced by substituents, including hydroxypropyl (HP) or sulfobutyl ether (SBE) groups (Figure 2). The European Medicines Agency (EMA) has approved the use of six CDs as excipients for use in the pharmaceutical industry, namely α-CD, β-CD, γ-CD, HP-β-CD, SBE-β-CD, and randomly methylated (RM-) β-CD [3]. In recent decades, complexes of organic molecules of the mentioned CDs have been of particular interest to researchers.

In the pharmaceutical industry, CDs are used as excipients at the preformulation stage to exploit the advantages resulting from the complexation of active pharmaceutical ingredients (APIs) with an appropriately selected host. There are many possible benefits from CD complex formation, including increased solubility [4], reduced degradation and decomposition of the API [5], enhanced permeability through biological membranes [6], modified drug-release profiles (immediate, sustained, or targeted) [7,8], and masking of the bitter taste. These advantages extend the use of CDs to the cosmetic and food chemistry industries [9]. When combined with specific class APIs, CDs can enhance their pharmacological effect against bacteria [10], viruses [11], inflammations [12], cholesterol [13], and cancer [13]. Currently, there are at least 130 pharmaceutical products containing CDs available on the market [1,14]. From an economic point of view, it is cheaper and faster to develop a novel CD-based formulation than to introduce a new API (which carried a cost of USD 2.3 billion in 2023 [15]). Moreover, CDs are regularly used in analytical chemistry, since they are known to improve the sensitivity of the spectrofluorometric method [16] and they allow for the separation of pairs of enantiomers [17]. In response to the ever-growing environmental concerns, CD-based nanosponges have evolved as important adsorbents for wastewater treatment, enabling the removal of various pollutants, such as heavy metals, organic dyes, phenols, and pesticides [18].

In conjunction with experimental research, computational techniques have been widely used to explain the formation mechanisms of inclusion complexes with CDs [19,20]. Thanks to molecular modelling techniques, it is possible to simulate the 3D structure of the complex being formed, indicate the interactions between the host and the guest, and justify its thermodynamic stability [19,20]. So far, five factors have been found to influence the driving force of CD inclusion complexations [21]: (i) van der Waals interactions; (ii) electrostatic interactions; (iii) formation of hydrogen bonds; (iv) relief of conformational strain in the CD molecule when the guest enters its cavity; and (v) exclusion of cavity-bound high-energy water. Other applications of theoretical research involve predicting the thermodynamic stability of CD complexes with previously unstudied organic molecules. Such predictions can, for example, show which CD will form the most stable complexes with a particular guest or what the best environmental conditions will be for complex formation. Therefore, it is very beneficial to precede expensive experimental studies with much cheaper computer-aided studies. This is especially true for certain drug molecules that are either in the clinical trial phase, hard to obtain, or entail unreasonably high synthesis costs.

The goal of quantitative structure–activity relationship (QSAR) analysis, also known as quantitative structure–property relationship (QSPR) analysis, is to develop an algorithm that estimates and predicts specific activities or properties of a new compound, knowing only the values of that property for other previously studied compounds. QSAR/QSPR methods are used when the theory of the phenomenon under study is unknown or very complex, making it challenging to construct a mechanistic explanatory model [22]. The success of the QSAR/QSPR approach is due to access to an increasing number of databases, as well as the use of complex machine learning (ML) methods that use data to create complex relationships between the variables. ML can be considered a component of so-called artificial intelligence (AI), which aims to imitate human capabilities such as learning, reasoning, and problem solving. The popularity of AI and ML approaches has allowed for a paradigm shift from traditional trial-and-error or hypothesis-driven methods to a data-driven approach [23].

An important application of QSAR analysis is drug development, where the affinity of a new drug candidate for a specified biological target is predicted based on results obtained for known drug molecules [24,25]. Thanks to AI, scientists identified a new powerful antibiotic in 2020 [26]. The ML model screened over 100 million molecules selected from the ZINC15 database [27], which is an online collection of approximately 1.5 billion chemical compounds. This analysis, which took just three days, identified 23 candidates that were structurally different from existing antibiotics and were predicted to be nontoxic to human cells. In laboratory tests on five species of bacteria, scientists found that eight molecules had antibacterial properties, two of which were found to be particularly active [26].

There are many other applications of AI and ML in chemistry and pharmaceutical sciences. The fundamental problem of predicting the 3D structure of a protein based solely on the amino acid sequence can be solved more quickly and efficiently by using an AI-assisted homology modelling tool such as AlphaFold [28]. The QSPR approach is of great importance in predicting the pharmacokinetic properties of new molecules (drug candidates), such as absorption, distribution, metabolism, excretion, and toxicity (ADMET) [23]. Further examples of the use of data-based algorithms are the prediction of the physicochemical properties of compounds, predicting chemical reactions, computer-assisted structure elucidation, analytical chemistry (chemometrics), agricultural research, cosmetics, as well as food, materials, and regulatory science [29].

In predicting the thermodynamic stability of CD complexes, each QSAR/QSPR method consists of three important elements: (i) a dataset, i.e., tabular experimental values of the analysed property measured for the compounds studied so far; (ii) a set of descriptors characterising the structure and physicochemistry of the molecules from the dataset; and (iii) an algorithm that quantitatively expresses the relationship between the descriptors and the property under investigation. Therefore, a model can be imagined as a mathematical function of molecular descriptors. The function contains many parameters, some of which can be treated as weights assigned to descriptors. The process of learning (training) the algorithm involves adjusting the parameters so that the difference between the predicted and experimental values is as small as possible for all selected compounds from the dataset. The predictive performance of a model is influenced by all three components: the dataset used, the descriptors, and the ML algorithm.

In 2011, Ghasemi et al. [30] published an exhaustive review of QSAR/QSPR papers with a focus on estimating the stability constants of complexes of macrocyclic molecules (CDs and crown ethers) with various guest molecules. Most of the discussed models of inclusion complex formation from 1979 to 2011 described only linear relationships between variables. However, recent significant advances in datasets, descriptors, and algorithms have made it necessary, in our opinion, to provide researchers with a novel, updated review, covering reports from the last decade. Moreover, review articles have recently been published discussing the achievements of other theoretical methods in relation to the formation of inclusion complexes using CDs, including quantum-chemical methods [31], molecular dynamics [32], and docking methods [33]. Hence, there is not only a need to provide updates and discuss the recent achievements of the QSAR/QSPR analysis methods but also to propose solutions on how to effectively use this knowledge, especially for those who will deal with this topic in the future.

Therefore, this review article discusses the use of QSAR/QSPR methods to predict the formation of CD complexes, analysing the achievements and results obtained between 2011 and 2023. Particular attention was given to the use of non-linear ML methods, which have revolutionised many aspects of everyday life. It is hoped that the use of ML algorithms will yield comparable success in predicting the properties of CD complexes. This article aims to serve as a bridge between chemists and software developers, allowing for more efficient cooperation between them.

## 2. Thermodynamic Stability Predictions of CD Inclusion Complexes

### 2.1. Theoretical Background

When CD and the ligand are dissolved in a medium such as water, a certain number of ligand molecules will enter the CD cavity and the system will reach chemical equilibrium, as described by the stability (association) constant K:

(1) $$K=\frac{\left[CD\cdot L\right]}{\left[CD\right][L]}$$

where the terms $\left[CD\cdot L\right]$, [CD], and [L] represent the concentrations of the complex, the free CD, and the uncomplexed ligand, respectively (all concentrations are expressed in mol dm^−3^). The resultant unit of K, mol^−1^dm^3^, which is often denoted as M^−1^, is usually omitted.

The larger the K value, the more guest molecules enter the CD cavity. In other words, the equilibrium is shifted towards complex formation. K is directly related to the Gibbs free energy, Δ*G*, which in turn can be considered as the driving force for the process:

(2) ΔG=−RTln⁡K

where R = 8.314 J mol^−1^ K^−1^ denotes the universal gas constant and T (K) is the temperature of the system. As can be seen from Equation (2), Δ*G* and ln⁡K are proportional to each other. In QSAR/QSPR studies, both Δ*G* and ln⁡K or $\log_{10}K$ predictions can be found, often based on the same dataset.

### 2.2. Datasets

The dataset is a tabular summary of the experimental values of Δ*G* and ln⁡K or $\log_{10}K$ determined for CD complexes with various guest molecules. Each individual pair containing the structure of the guest and corresponding measure of the complex thermodynamic stability is called a data point. The quality of the developed models is inextricably linked to the quality of the datasets used. An algorithm trained on poor data results in poor predictions, even if the evaluation metrics are satisfactory.

Currently available datasets are combinations of previous compilations of data, often determined using different methods. There are many experimental techniques for determining the thermodynamic stability of CD complexes [34], including phase solubility studies [35] using ultraviolet–visible (UV–vis) spectroscopy or high-performance liquid chromatography (HPLC), fluorescence spectroscopy, nuclear magnetic resonance (NMR), and isothermal titration calorimetry (ITC). Importantly, due to differences in experimental conditions, significantly different values may be obtained for the same complex [1,34]. In 1998, Rekharsky and Inoue [36] published a compilation of thermodynamic data for complexes of 1:1 stoichiometry with 3 native (α-, β-, γ-) and 30 substituted CDs, which were obtained using 14 different techniques. For each guest compound, the following values are provided: log⁡K, Δ*G*, the change in enthalpy (Δ*H*), and entropy (Δ*S*), along with their measurement uncertainties (if available); the name of the experimental technique; and a description of the solvent, including pH and temperature. When more than one result was found in the literature for the same compound, all individual measurements were presented, and the results were not averaged. This approach facilitates the assessment of the comparability of values obtained from different measurement techniques, the identification of potential outliers, and the determination of whether averaging all results for each guest is justified. Surprisingly, this dataset has rarely been used in QSAR/QSPR modelling for CD complexes [37,38,39]. Future reports may include the measurement technique by introducing an additional feature (descriptor) into the created ML model.

However, other available datasets often lack information on the experimental measurement technique. Nine papers discussed in this review are based on a similar dataset, including thermodynamic stability measures for the complexes of β-CD with more than two hundred guest molecules; however, different researchers cite different papers as the source of this data. Suzuki [40] was the first author to collect a 218-item training dataset and a 15-item test set from 10 different literature sources containing the values of ΔG of complex formation. The 218-item training dataset for β-CD was then used in a subsequent study by Katritzky et al. [41], which is also often cited as an original source. Pérez-Garrido et al. [42] presented the same data as Suzuki [40] but with combined training and test sets, i.e., containing 233 items, and expressed in the form of decimal logarithms of K. However, the discussed dataset does not provide direct information about the experimental conditions of determination of K. Furthermore, it is uncertain whether these values were obtained using the same measurement technique.

An alternative approach was proposed by Zhao et al. [43], who, based on reports from 1990 to 2018, collected a 3000-element dataset of Δ*G*s determined solely by the phase solubility study method [35]. This procedure prevented errors that would result from differences in measurement conditions when using different experimental techniques. However, this particular dataset has not been made publicly available. Another noteworthy dataset was collected using the ITC technique for complexes of 43 guests’ enantiomer pairs with β-CD [44]. Generally, these data may be utilised by any model to perform an additional external test to verify whether the developed method correctly describes the phenomenon of chiral recognition using CDs.

In recent years, due to the ever-increasing number of experimental results, a lot of effort has been put into building large datasets. Mizera et al. [45] searched and found 8534 records of ln⁡K from the web database cyclodextrin.net (a database that is no longer available at the time of writing this article). The data curation process was performed according to the recommendations from [46,47] and consisted of the following steps: (i) removing records for the compounds without clear structural information; (ii) merging or removing duplicates (the data for the same host and guest under the same conditions); (iii) selecting compounds with molecular weights in the range of 50–500 Da; and (iv) selecting the measurements carried out at temperatures from 20 to 30 °C and in a pH range of 5–8. This procedure reduced the number of data points to 1654, with the resulting dataset published in the Supplementary Material of the work [45].

In 2023, a curated dataset of CD–guest association constants was reported [48], gathering the literature values from the period 1963–2021, including the datasets mentioned above [36,40,45], as well as others [49,50,51,52,53]. The authors retrieved data directly from PDF files containing relevant articles and performed a multi-step data curation process. The evident outliers of thermodynamic values were removed. If ΔG/log⁡K for the same CD–guest pair differed between sources, values obtained by a more accurate technique were selected, or values were compared to those obtained for structurally similar molecules. The following measurements were discarded: (i) pH outside the range of 6.9–7.4, (ii) temperatures outside the range of 14.5–30.1 °C, (iii) solvents other than water. The raw dataset and its enriched version (after pre-processing and calculation of some descriptors) were made accessible to the scientific community on the Zenodo platform [54]. The access to publicly available data, containing 3459 results obtained for 15 different CDs and 1767 guest molecules, will hopefully open new possibilities in the field of ML development.

### 2.3. Descriptors

In QSAR models, it is necessary to express the structural and physicochemical properties of a molecule in the form of numeric values known as descriptors. There are many computer programs to calculate them, including PaDEL [55], DRAGON [56], or Molecular Operating Environment (MOE) [57], meaning it is possible to obtain several thousands of descriptors for each compound in the dataset. The suppliers of the software provide the users with a detailed list of individual descriptors together with their definitions. Hence, only selected types of descriptors are discussed below rather than presenting individual ones.

The molecular structure of a guest can be represented using the simplified molecular input-line entry system (SMILES). The SMILES string consists of upper and lower case letters, numbers, and symbols, e.g., the SMILES code CCC(=O)O describes propionic acid, and Cc1ccccc1 represents toluene. For molecules with stereogenic centres, the structure can be described using the isomeric SMILES notation, in which the symbols @ and @@ denote two different arrangements of substituents at the stereocentre (clockwise and anticlockwise). For example, the isomeric SMILES code C[C@H](C(=O)O)N denotes D-alanine, whereas C[C@@H](C(=O)O)N describes L-alanine. There are many approaches to convert the alphanumeric SMILES notation into numeric values, which are available to ML algorithms. The novel Iso2vec model is able to convert an isomeric SMILES into a set of ten real numbers, functioning similarly to another state-of-the-art algorithm known as word2vec [48]. As a result of Iso2vec treatment, similar molecules should be described with a set of similar numbers.

An alternative strategy for describing molecular topology is to divide the structure of a molecule into fragments, each containing several atoms. Structural keys and molecular fingerprints are sequences of bits carrying the numbers 0 or 1. Each bit corresponds to the presence (1) or absence (0) of a substructure from a predefined list. To enable the most accurate predictions of CD complex formation, Di et al. [58] compared five different fingerprints describing the structure of guests. Among them, the Klekota–Roth fingerprint was found to be appropriate for describing α-CD complex formation, while the PubChem fingerprint was found to be suitable for predicting complexation with β-CD [58]. Another study determined the optimal descriptor as a function of the SMILES notation of molecular fragments [59]. The Monte Carlo method was used to determine the values of weights assigned to individual substructures.

To calculate topological descriptors, also known as 2D descriptors, the molecule is treated as a graph consisting of atoms and the chemical bonds connecting them. Norm indices [60,61] are calculated using both step matrices, expressing various interatomic connections, and property matrices, characterising the properties of individual atoms. Niu et al. [61] stated that for α-CD complexes, three of the five most important norm indices corresponded to the number of the outermost electrons, while for β-CD complexes, three of the five most significant norm indices corresponded to the number of branches in the molecule.

According to the review by Ghasemi et al. [30], the QSAR/QSPR models developed up to 2011 are mainly based on topological descriptors, which have certain limitations related to the lack of sufficient parameters describing drug–CD interactions and the inability to represent the stereochemistry of the guest molecules. On the other hand, the calculation of 3D descriptors requires knowledge of the three-dimensional geometrical structure of the studied molecules, which is obtained from 3D X-ray crystallographic structural data or theoretical calculations of the optimal geometry. In recent years, thanks to easier access to computational power, many researchers have begun their QSAR/QSPR studies by optimising the geometries of all the guest molecules from the dataset before calculating 3D descriptors using the obtained structures. For example, in the model of Sang et al. [62], a set of structural descriptors was derived from the electrostatic potentials determined on the molecular surface. Meanwhile, Jeschke and Cole [63] developed a method to predict β-CD complexation using the set of spectrophores, which constitute another set of 3D descriptors. To compute them, the molecule is rotated in a virtual cage, and its interaction energy with the cage is calculated by sampling the orientation of that molecule in angular steps [64].

In 2017, Solovev and Solov’ev [65] noted that previously developed ML algorithms did not focus on correctly predicting Δ*G*s for guests that were optical isomers. CDs are chiral; therefore, their complexes with chiral guests are diastereosiomeric, usually with varying stability. However, when QSPR models are based only on topological 2D descriptors, then identical thermodynamic properties are predicted for the complexation of antipodal guests [66]. To solve this problem and to enable the prediction of chiral recognition, the authors developed a new computer program called *mfSpace* (molecular fragment space), which aims to generate and code 3D fragment descriptors. In this way, the authors managed to predict different free energies for antipodal guests from a specialised dataset, as discussed in a previous subsection [44]. However, subsequent reports have not tested chiral recognition as part of the assessment of the algorithm’s predictive performance.

Another popular approach in QSAR is known as comparative molecular field analysis (CoMFA). Using this method, the 3D structures of the studied molecules must first be aligned in space. Then, the molecular interaction fields (MIFs) are generated for each grid point surrounding the molecule, typically including electrostatic and steric fields, with the latter being directly related to the shape of the molecule. Another set of MIFs, used in the model of Linden et al. [67], consists of quantum mechanically based local sigma profiles derived from the COSMOsar3D method using a 3D grid of COSMO-RS (conductor-like screening model for real solvents) surface polarisation charge densities. Generally, the number of calculated MIFs often significantly exceeds the number of data points in the dataset, and the MIFs determined for a given point are highly correlated with MIFs at neighbouring points. To solve these problems and make the analysis possible, a special mathematical treatment called the partial least squares (PLS) method is used in model development. However, the CoMFA method has two limitations: (i) the correct conformation of the molecule must be used, which may not be the lowest-energy conformation, and (ii) the compounds must be properly aligned, which is a time-consuming process that may introduce errors [68]. To overcome these limitations, Ghasemi et al. [37,68] used a set of grid-independent descriptors (GRIND) in the predictions of β-CD complex formation, enabling the assignment of significant importance to variables describing the size match between the CD cavity and the guest. Another method for calculating alignment-independent descriptors is known as VolSurf, which has been used to predict the stability of α-CD complexes with 72 monosubstituted and 1,4-disubstituted benzenes [69].

A common approach in QSAR/QSPR predictions for CD inclusion complex stability is to generate the maximum possible number of descriptors using software from various vendors and then reduce their number using appropriate unsupervised ML algorithms. For example, Cysewski et al. [70] used the ChemDes platform [71] to obtain 3679 various descriptors from different software, including PaDEL [55] and ChemoPy [72]. Among the papers discussed in this review, the featured selection methods were stepwise multiple linear regression [60,73,74,75], genetic algorithm [37,69,70], principal component analysis [73,76], successive projections algorithm [69], fractional factorial design [68], autoencoders [39], as well as backward [58] and recursive [38] feature elimination algorithms. Due to the continuous development of ML theory and practice, the trend of using advanced feature selection algorithms is likely to continue. Many of the cited works contain a list of several descriptors that are most important in the model being developed. It turns out, however, that such a list is not universal, as each paper contains a different selection of the most important descriptors. Moreover, the physical meaning of individual descriptors is often not obvious and is difficult to interpret.

Typically, a separate model is developed for each individual CD, and then the descriptors are calculated only for the guest molecule. Recently, a slightly different approach has been used, in which descriptors are calculated not only for the guest but also for the host [43]. This led to the development of one model covering several different CDs and the construction of larger datasets, including up to 3000 data points [43]. The descriptors calculated for hosts and guests are accompanied by experimental conditions, such as pH and temperature. However, the distribution of CDs in the available datasets is not uniform; β-CD is the most common, while less common CDs, such as trimethyl-β-CD, are represented by a small fraction of data points. One may suspect that the model would be more robust for approved CDs and less predictive for atypical hosts. Therefore, it would be good practice to calculate evaluation metrics for each individual CD (as in [53,77]) rather than providing only one set of measures averaged over the entire dataset. In this way, an appropriate measure of uncertainty could be determined for each CD and then applied to accompany the predictions for guests from outside the dataset.

Particular attention should be paid to randomly substituted CDs, such as HP-β-CD, SBE-β-CD, and RM-β-CD. In fact, they are mixtures of various compounds in which hydroxyl groups are substituted to a different extent with appropriate substituents (HP, SBE, and methyl). Therefore, these CDs are only characterised by a specified average degree of substitution (DS). Meanwhile, some authors have reported the development of single algorithms covering both native and substituted CDs. However, the process of generating the descriptors for randomly substituted CDs is then not clearly described. In the future, when a dataset is collected for any randomly substituted CD, it would be beneficial to ensure that the CDs of the same type are characterised with the same DS. An interesting approach to substituted CDs was presented by Merzlikine et al. [78], who collected a novel dataset for a single CD known as Captisol (SBE-β-CD characterised with an average DS of 6.5), containing 220 complexation constants determined by the phase solubility study method [35]. An important advantage of developing an ML model for complexes exclusively with Captisol is that, in this case, no knowledge of the chemical composition of this SBE-β-CD is required.

Another interesting paper assumed that the stability of the complex with β-CD does not only depend on the molecular properties of the guest but also on the effects of conformational reorganisation, which should also be taken into account [75]. For this reason, the guests were docked into the β-CD cavity, and the lowest-energy conformation was selected for each complex to calculate the 3D descriptors (geometrical, energetic, and electronic) related to the binding between the host and the guest. Additionally, some descriptors for single ligands were calculated but they were based on the conformations of guest molecules extracted from the inclusion complexes [75].

### 2.4. ML Algorithms

In general, predictive ML algorithms can be divided into two distinct classes. If the dependent variable, *y*, can take any numerical value from the selected range (this applies to K, log⁡K, and Δ*G*), they are known as regression models. Model development is based on minimising the difference between the predicted *ŷ* and the experimental *y* from the dataset. In turn, classification models are based on datasets in which the *y* value denotes the group that the data point belongs to, e.g., whether or not the complex has formed.

In the most basic model, known as multiple linear regression (MLR), the *y* variable (e.g., Δ*G*) is expressed as a linear combination of the *x* variables (such as molecular descriptors). Among the QSAR/QSPR models discussed in this review, MLR was used in [60,61,62,68,75]. The process of training the MLR algorithm involves finding optimal values of weights, $w_{i}$, assigned to individual descriptors, $x_{i}$. Generally, the obvious advantage of the MLR model is the ability to write a basic equation showing a simple direct relationship between variables (here $\Delta G=w_{1}x_{1}+w_{2}x_{2}+\ldots$), which is impossible or impractical to do with most other ML techniques. Often, researchers additionally try to find the appropriate number of descriptors included in the model. In the stepwise MLR approach used in [73,74], descriptors are added to the equation step-by-step, and a new regression is performed each time.

A substantial limitation of the MLR algorithm is the ability to find only linear relationships between variables, which is inaccurate in the case of a large dataset comprising structurally various chemical compounds. Due to this restriction, as well as its much longer history, MLR is considered a statistical model rather than an ML method. Nevertheless, many different ML models have been proposed to find non-linear relationships connecting the *y* variable with individual *x* variables and their combinations. Most of the models discussed below are the so-called ‘black boxes’, which means that despite high prediction accuracy, it is difficult to trace the calculations made by the model and draw general conclusions about the relationships between variables. A detailed mathematical explanation of the models discussed, among others, can be found in the indicated handbooks [79,80].

Support vector machines (SVMs) are a valuable technique useful in both classification and regression problems. In the latter case, support vector regression (SVR) is able to find both linear and non-linear relationships between variables. In the context of predicting CD complex formation, this technique has been used in [53,62,69,77,81]. To solve non-linear problems, the so-called kernel functions are used, including the Gaussian radial basis function (RBF) and the polynomial and sigmoid kernels. Additionally, a slightly different mathematical treatment of the problem exists, known as least squares SVR, which was used in some studies of CD complexes [60,62].

**Figure 3:**
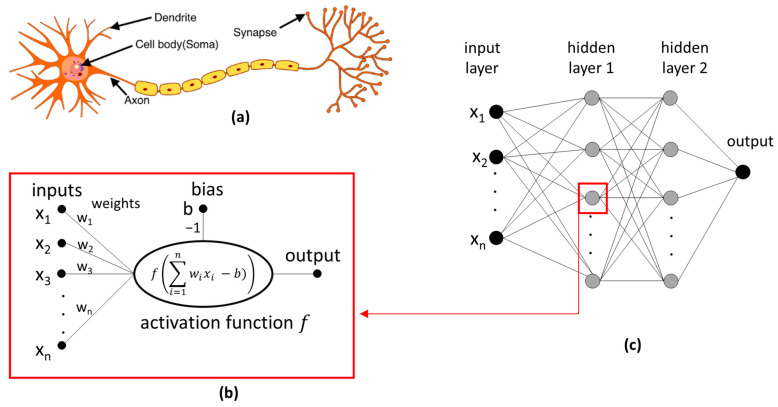
(**a**) Biological neuron (adapted from [82]), (**b**) artificial neuron, and (**c**) artificial neural network.

Artificial neural networks (ANNs) play a very important role in ML. The concept of an ANN reflects the functioning of the human brain, which consists of many neurons. In biological neurons (Figure 3a), input signals are collected by dendrites and then processed by the soma, while the output signals are transmitted through synapses to subsequent neurons. Meanwhile, the artificial neuron calculates the weighted sum of inputs and a non-linear function of the obtained result before transmitting the result to the next layer of neurons (Figure 3b). The values of the weights result from network training and represent the influence of the input on the output, i.e., the strength of the connection between two neurons. The ANN consists of several layers, as shown in Figure 3c: (i) an input layer, with each neuron corresponding to a single descriptor; (ii) a hidden layer(s); and (iii) an output layer, corresponding to the calculated result (e.g., Δ*G*). An ANN with one hidden layer to predict CD complex formation was used in [60,73]. An ANN that has more than one hidden layer is often called a deep neural network (DNN), with the technique commonly referred to as deep learning (DL). The examples of the DL models discussed in this review include references [39,43,76].

Another approach in ML is the use of decision trees (DT). Each DT consists of branches connecting the nodes. In the nodes, certain conditions are checked for an analysed compound, often formulated as an inequality related to one of the features, e.g., ‘molecular weight < 1000 Da’ or ‘octanol/water partition coefficient log⁡P < 0′. Depending on the answer, the algorithm moves on to the next node, where the next condition is checked. Therefore, single DTs are easy to interpret. Finally, the algorithm reaches the appropriate leaf. For classification purposes, the leaf contains the label of the appropriate class. In a regression tree, each leaf represents the mean of the dependent variables for all data points from the training subset that were assigned to the same leaf. The Cubist model (used in [38,78] to predict CD complex formation) can be considered as a DT in which each leaf contains an equation of a linear regression instead of a single value of the *y* variable [83]. To improve the prediction accuracy, multiple independent DTs are trained using a randomly sampled subset of the dataset and randomly selected variables, thus creating an ensemble model called a random forest (RF). The RF model is widely used to predict CD complex stability [38,43,58,62,63,78] but the action of the model is difficult to interpret.

It is possible to combine many different models into one ensemble model. For example, predictions calculated by different algorithms can be averaged. When several models run sequentially, it is referred to as gradient boosting (GB). The first model makes predictions for compounds from the dataset, and then the differences between the experimental and predicted values are calculated. These errors become *y* variables for the next algorithm. The procedure is repeated until the errors are acceptably small. Examples of GB algorithms include the gradient-boosting machine (GBM) [38], LightGBM (an open-source gradient boosting tree algorithm by Microsoft) [43,45], extreme gradient boosting (XGB) [53], or gradient-boosted trees (GBT) [58].

Other ML regression algorithms used in the cited studies include multivariate adaptive regression splines (MARSplines) [38,70], Gaussian process regression (GPR) [53,58,62], and radial basis function (RBF) regression [58]. It is worth noting that the construction of ensemble models may consist of many optimisation steps. For example, in the work of Di et al. [58], initial ML models were developed using the following algorithms: RBF regression, GPR, RF, GBT, and tree ensemble. Following this, the so-called consensus models were constructed, each considering a different combination of initially optimised models. The predicted K of the consensus model was defined as the weighted average of the K values obtained in the single models, while the weighing factor was the *R*^2^ value of the single model.

In many reports, several ML algorithms are independently optimised based on training data and then their predictive capabilities are compared. This approach can be justified by the very important ‘no free lunch theorem’, which states that no single ML algorithm exists which is universally most effective for all problems [84]. Depending on the dataset used and the calculated set of descriptors, different algorithms performed better in different reports. In the case of CD complexation predictions, the authors obtained the best predictive performance using GPR [53], least squares SVR [60], RBF regression [58], ANNs [73], and LightGBM [43]. Therefore, an interesting question arises: how will the statistical metrics of models developed before 2023 change when the same algorithms are optimised, for instance, by using a larger, publicly available dataset [48]?

As mentioned earlier, despite the increasing accuracy of predicted values, it is still a great challenge to develop an ML model that is easy to interpret and allows for drawing general conclusions. This is because ML models are often very complex mathematical functions of features, often being non-linear functions of combinations of descriptors. As discussed in Section 2.3, many authors reduce the number of descriptors involved before optimizing the model. Feature selection techniques select only descriptors of values that vary significantly across compounds from a dataset, enabling the development of simpler ML models. However, this approach does not allow for estimating the impact of individual variables on predicted value.

In order to make the model interpretable, a list of input variables with the greatest impact on an output variable must be determined. The methods of ranking the feature importance differ depending on the ML model used. For linear models, such as MLR and PLS, the effect of $x_{i}$ on y can be easily determined from the regression coefficients. The stepwise MLR method further finds the optimal number of included descriptors, thus avoiding under- and overfitting. DT and RF models have an intrinsic ability to estimate the importance of the future. Namely, some descriptors are considered significant if they are used on single DT nodes and effectively partition data points according to the results of mathematical inequalities containing them. In the case of neural networks, determining important features affecting the CD complexation phenomenon is possible thanks to the development of novel attention-based DNNs, such as AttPharm [81]. Additionally, the use of two post hoc models enables the interpretation of any already developed ML model. Shapley additive explanations (SHAP) are able to estimate the relative importance of relevant descriptors, while local interpretable model-agnostic explanations (LIME) provide further information on whether the impact of the analysed feature on Δ*G* is positive or negative [81].

### 2.5. Model Validation

According to the guidelines published by the Organization for Economic Co-operation and Development (OECD) [85], a QSAR model should be associated with the following information: (i) a defined endpoint; (ii) an unambiguous algorithm; (iii) a defined domain of applicability; (iv) appropriate measures of goodness-of-fit, robustness, and predictive performance; and (v) a mechanistic interpretation, if possible.

When evaluating a model, it is very important that the dataset contains chemicals that exhibit a continuum of physicochemical properties. The presence of outlying points, i.e., compounds with significantly different properties compared to the rest of the dataset, would have a negative impact on the developed model and reduce its predictive power. To check for their existence, the applicability domain (AD) of the model is determined [85]. For each compound in the dataset, the leverage, $h_{i}$, is calculated and compared to the warning leverage, $h^{*}$. Determination of the AD of the model should provide a list of the outlying compounds, i.e., those for which $h_{i}>h^{*}$.

A good ML model should not only predict the correct values for compounds in the dataset but it should also be able to make accurate predictions for new compounds from outside the dataset. However, there are two opposite deviations from optimal performance that may occur with the developed algorithms. In the first case, when the model is too simple and is unable to properly describe the analysed phenomena, it is known as an underfitted model. The second case occurs when the model is too complex, e.g., it involves too many descriptors or uses mathematical functions with too many weights. Then, predictions for compounds from the dataset would be unrealistically accurate given the experimental measurement uncertainties of the *y* values, while predictions for new compounds would be significantly less accurate. These are termed overfitted models.

**Figure 4:**
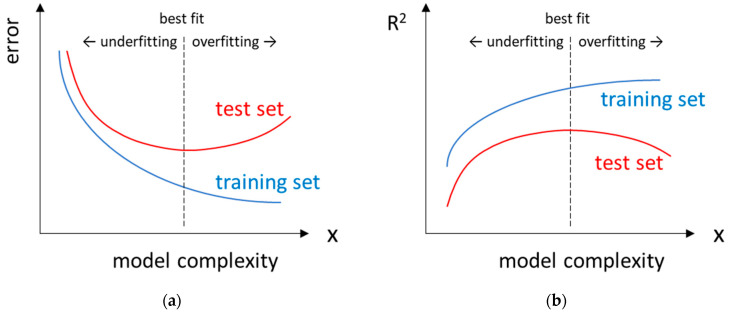
(**a**) Prediction error and (**b**) coefficient of determination, *R*^2^, as a function of model complexity plotted for data from the training and test sets. Part (**a**) is adapted from [86].

There is a generally accepted strategy for checking whether models are under- or overfitted. Before training the model, the dataset should be divided into at least two subsets, namely the training and test sets. Then, several models varying in complexity (e.g., the number of used descriptors) are developed using only the data from the training set, which means that at the training stage, the model ‘does not know’ about the existence of the test set. The test set is used to evaluate the optimised models. The prediction error is then plotted against the complexity of the model (Figure 4a). In the case of underfitted models, large errors occur in both the training and test sets. On the other hand, an overfitted model fits the training set too closely (unrealistically small error) and is therefore unable to make relatively good predictions for the compounds in the test set (resulting in a large error).

It is worth mentioning that when comparing several algorithms, it is good practice to separate an additional validation subset [87]. This subset should be used to select the best algorithm, while the test subset should serve as a means of final evaluation of the model. Using this approach is particularly important when training neural networks, optimising hyperparameters of the created ML models, or performing feature selection. Otherwise, there is a risk of choosing a model that achieves particularly good performance when dealing with data from the test set. However, this recommended approach of dataset splitting into three parts is rarely found in the articles discussed (with noteworthy exceptions in [38,43,59,81]). For small datasets where there is a danger that a validation subset would not be large enough, an alternative reasonable approach to model selection can be applied. It involves cross-validation of the model, which is discussed in detail in the next subsection.

#### 2.5.1. Validation of Regression Models

Typical evaluation metrics used for regression analyses are defined in Table 1. The coefficient of determination (*R*^2^) can be thought of as the goodness-of-fit for a straight line fitted to a plot of predicted values against experimental values. According to Figure 4, as the complexity of the model increases, the *R*^2^ value calculated for the training set (*R*^2^_train_) will increase continuously, while for the test set, *R*^2^_test_ reaches its maximum value and then decreases because of overfitting of the model.

An additional recommended step in model evaluation is cross-validation (CV), which is performed using data from the training set. In the articles discussed, the most common procedure was the leave-one-out (LOO) CV. For each compound, the model was trained separately using all remaining data points and the corresponding *y* value was predicted. Based on the comparison of the predicted and experimental *y* values, the cross-validated correlation coefficient, *Q*^2^_LOO_, is calculated using the same general formula as one used for the determination of *R*^2^. However, performing LOO CV is only feasible for relatively small datasets because this approach requires as many optimization steps as there are data points. In the reviewed articles, the largest dataset for which LOO was performed contained 330 compounds.

In general, LOO CV can be considered as a special case of *k*-fold CV, where *k* is equal to the number of examples in the dataset. In any *k*-fold CV, the training set is divided into *k* subsets and the following procedure is repeated *k* times: one of the subsets is excluded from the dataset and the algorithm is trained on the remaining examples. Therefore, the smaller the value of *k*, the fewer optimisation steps need to be performed. For example, the papers reviewed in this work include CV performed using leave-two-out [67] and leave-ten-out [60] approaches.

In the assessment of predictive performance, it is recommended for both the training and test sets that *R*^2^ should exceed 0.6 and that *Q*^2^ should be greater than 0.5 [87,88].

To enable a direct comparison of the developed models, the most important information from the individual papers discussed is summarised in Table 2 (for α-CD), Table 3 (for β-CD), and Table 4 (for SBE-β-CD and single models covering several CDs). Metrics such as the mean absolute error (*MAE*) or the root mean square error (*RMSE*) depend largely on how the *y* value is expressed (in the form of Δ*G*, ln⁡K, or $\log_{10}K$). To enable comparisons between different models, in this review the metrics for ln⁡K and log⁡K have been converted so that they correspond to Δ*G* (expressed in kJ/mol).

The predictive ability of the model is simultaneously influenced by the dataset used, the set of descriptors, and the ML method. Therefore, it is difficult to study the effects of each constituent individually. Among the predictions of the thermodynamic stability of complexes from 2011 to 2023, it is an extremely difficult task to identify one algorithm with the best performance. Firstly, the discussed models use different datasets, including a different number of compounds and different extents of chemical diversity. An algorithm trained on a broader group of chemicals is unlikely to achieve an *R*^2^ value similar to a model describing only a selected set of structural analogues, such as disubstituted benzenes, but would be more suitable for making predictions for any compound from outside of the dataset.

It would be much easier to compare reports if the same criteria of method evaluation were used. As shown in Table 2, Table 3 and Table 4, different authors use different metrics to evaluate their models. Of the five common parameters (*R*^2^_train_, *R*^2^_test_, *Q*^2^_LOO_, *MAE*_test_, and *RMSE*_test_), only some selected values are determined and clearly described in individual papers. In the future, there is a strong need to use uniform, generally accepted, and clearly defined nomenclature. Currently, some authors perceive ‘external validation’ as making predictions for a test set, while other researchers understand the term as an additional test using compounds from outside the entire dataset. It is very important to check the AD domain of the developed methods. As shown in Table 2, Table 3 and Table 4, this treatment was included in less than half of the cited studies and was almost completely omitted in the models covering multiple CDs. Due to the ever-increasing size of available datasets, it is expected that LOO CV will no longer be a reasonable way to conduct CV, and researchers will switch to alternative methods, such as 5- and 10-fold CV.

Additionally, analysis of Table 2, Table 3 and Table 4 shows that many different algorithms were used in the discussed works to divide their respective datasets into training and test sets. Selecting the correct splitting algorithm can have a critical impact on the relationship between R^2^_train_ and R^2^_test_. Moreover, the recommendation to compare different models using an additionally separated validation subset is often not followed, which may lead to the selection of a model that is overfitted to the test subset and does not cope well with data from outside the dataset. Another strategy was also found, in which a random division of the dataset was repeatedly performed, thus obtaining many different variants of the split [38].

Based on the evaluation metrics, Kerner and von Recum [39] appear to have achieved the best results, i.e., an *R*^2^_test_ value of 0.996 and an *MAE* value of 0.77 kJ/mol. In this preprint, two independent sets of descriptors were computed—one for the guest and one for the CD. To reduce their number, autoencoders were used for the first time in the study of CD complexes. The entire model consisted of two input layers (separately for host and guest), two encoders, and a dense DNN that was trained to predict Δ*G*. The optimal design of this DNN consisted of three layers, two with 100 hidden neurons each and one with a single neuron. However, the methodology is not entirely clearly explained, especially as there is no mention of cross-validation. According to the authors, the method can be considered a proof-of-concept model, but its use may currently be limited.

As summarised in Table 2, six reports related to α-CD complexes have been published since 2011. Among them, the best prediction ability is obtained by using a 229-element (the largest) dataset with norm indices as descriptors (*R*^2^_test_ = 0.93, *MAE*_test_ = 1.03 kJ/mol) [61]. For β-CD, Table 3 presents 14 regression models from 2011 to 2023. Nine of them used the Suzuki dataset [40], which is also attributed to Katritzky [41] and Pérez-Garrido [42]; therefore, the evaluation metrics of these selected models can be mutually compared. Among them, the only algorithm allowing an error of less than 1 kJ/mol was developed using DRAGON descriptors, stepwise MLR to select the seven most important features, and the optimised ANN to predict Δ*G*, achieving *R*^2^_test_ = 0.96 and *MAE*_test_ = 0.93 kJ/mol [73].

#### 2.5.2. Validation of Classification Models

The assessment of classification models is significantly different and is based on the analysis of a confusion matrix (Table 5). Based on the correctness of the assignments predicted by the model, the number of true positive (TP), false positive (FP), true negative (TN), and false negative (FN) cases are counted. Then, various evaluation metrics can be calculated based on these four numbers from Table 5, ranging from 0 (bad performance) to 1 (excellent performance). The equations in Table 5 show that the method avoiding FP predictions is characterised by high precision, while the model with a small number of FN assignments is characterised by high recall. When constructing a dataset for classification studies, it is extremely important to include a similar number of positive and negative cases. On the other hand, an imbalance in the preparation of the dataset leads to difficulties in training the model, and then the evaluation metrics do not correctly describe the predictive performance.

An interesting classification study was published by Ma et al. [76], in which the authors performed cooling crystallisation experiments to create a new dataset consisting of exactly 100 positive and 100 negative data points. The optimised ANN, SVM, and logistic regression (LR) algorithms were combined to create ensemble models that can minimise misclassification of two different types: a recall-first strategy aimed at avoiding FN assignments and a precision-first strategy focused on avoiding FP cases [76].

### 2.6. Comparison of QSAR with the Possibilities Offered by Molecular Modelling

In computational chemistry, one of the most profound concepts is the compromise between the accuracy of the results and the computational cost of obtaining them. Depending on the available computational power, researchers can choose from many techniques that differ in their degree of approximation. The methods of molecular modelling, which aim to obtain the 3D structure of a CD complex, are described below with increasing simplification and, consequently, decreasing accuracy.

Quantum-chemical methods are based on the numerical, approximate solving of the Schrödinger equation, which is the fundamental physical principle that governs the behaviour of electrons and atomic nuclei. While they offer the most accurate results, they are very time-consuming when applied to macromolecular systems like CD complexes. Additionally, the equations are solved iteratively, which means that the calculations continue until the results do not significantly change, making it difficult to estimate the time needed to perform the calculations in advance.

In molecular mechanics, a more generalised view of a single molecule has been proposed, in which atoms are treated as balls carrying certain partial charges and chemical bonds are imagined as springs with defined spring constants. The forces acting between atoms are calculated using the laws of classical physics according to the selected force field. In addition to the forces associated with chemical bonds, van der Waals and electrostatic forces are determined, including both intra- and intermolecular interactions. It is therefore possible to determine the energy of interaction between the host and the guest. Simulations of the studied systems commonly assume the presence of water molecules and extend the apparent size of the system by using periodic boundary conditions. This approach facilitates the understanding of the effect of the aqueous solvent on the formation of the CD complex. Using the method known as molecular dynamics (MD), the behaviour of the system is simulated as a function of time, showing the limited motions of guests inside the CD cavity. An important application of MD is the visualisation of the structures of complexes with two different enantiomeric forms of the guest, allowing for the assessment of the differences in their thermodynamic stability [17,95]. This knowledge may facilitate the process of selecting optimal conditions (e.g., solvent and pH) for the separation of enantiomers in capillary electrophoresis [17].

The use of molecular modelling studies can complement the developed ML model with a mechanistic explanation for one selected system. For example, MD studies explained the favourable solubility of a ternary system consisting of hydrocortisone, β-CD, and hypromellose [96]. It was found that hypromellose wraps around hydrocortisone and prevents the self-aggregation of β-CD molecules [96]. It is also worth mentioning that the values of Δ*G* predicted by QSAR/QSPR models for compounds from outside the dataset can be verified not only using experimental techniques but also using MD (as in [43]) or via a quantum mechanical approach.

Molecular docking also uses the concept of a force field, defining how forces between atoms are calculated. To find the preferred structure of the complex, the ligand (drug molecule) is moved, and the interaction energy with the receptor (CD molecule) is calculated for each arrangement using a scoring function. This approach allows for a very quick determination of the most probable structure of the complex. However, because of the simplicity of the scoring function, the obtained affinities are highly inaccurate compared to the results obtained using the two previously mentioned methods. Interestingly, the overall performance of the optimised QSAR models can be easily compared with the results obtained by other computational methods using the *R*^2^ metric. Rivera-Delgado et al. [38] performed molecular docking studies for compounds from the same dataset and plotted the calculated Δ*G* using the scoring function against the experimental Δ*G* values. The *R*^2^ value obtained in this way was significantly lower compared to the *R*^2^ value for an ensemble of ML algorithms, showing that the predictive performance of molecular docking was inferior to that obtained with an optimised QSPR model.

Compared to the molecular modelling approach, it can be stated that interpretability is still not a strong point of ML models. Although many studies identify a few (mainly five to eight) important descriptors, such a list is not reproducible across publications, and the meaning of many descriptors is neither straightforward nor intuitive. Moreover, most non-linear ML models (SVR, RFs, ANNs, and their ensembles) act as black boxes, making it difficult to clearly express the relationship between variables. On the contrary, quantum mechanics, MD, and docking studies allow us to obtain the most probable 3D shape of a complex and identify particular groups of atoms that interact with each other. Therefore, in terms of interpretability, computational chemistry methods can be considered superior to QSPR models when applied to CD complexes.

On the other hand, it should be noted that the molecular modelling methods estimate Δ*G* based on the analysis of a single 1:1 complex in water compared to single host and single guest molecules in an aqueous environment. However, such an approach may turn out to be a significant simplification, as it is widely known that hosts, guests, and complexes can form aggregates in aqueous solutions [1]. Meanwhile, QSAR/QSPR models are free of simplifying assumptions because the only information they require is the structure of individual guests, along with the measure of the thermodynamic stability of their complexes with a selected CD. Furthermore, there are certain cases where the use of the QSAR approach may be particularly beneficial compared to other computational methods. An important future application of ML may involve predictions for randomly substituted CDs, which are mixtures of different compounds. In molecular modelling, a common approach to this problem is to perform calculations on just one representative, averaged structure of the substituted CD. On the other hand, ML techniques do not require knowledge of the exact chemical composition of the CD being analysed, as they take advantage of experimental Δ*G*s measured for complexes of different guests with the same CD. The same reasoning applies to the modelling of CD complexation for guests with multiple ionisable groups across a wide range of pH. When using a standard molecular modelling approach, one should predict the pK_a_ value for each functional group (which is effectively accomplished with the use of ML), determine the dominant protonation state at a given pH, and perform a simulation using the selected species. Meanwhile, the ML algorithm may learn directly from the known pH-dependence of Δ*G* for various compounds and thereby predict the Δ*G* of a new compound at a specified pH. Naturally, to achieve this goal, appropriate datasets must be created, including data for randomly substituted CDs and for the pH-dependent variation in Δ*G* for compounds with ionisable functional groups.

An important goal of developing new QSAR algorithms should be to enable the scientific community to make novel predictions. Using the already developed ML method, calculations should be faster compared to quantum mechanics and MD. For this purpose, the scientific community should have access to all the information about the model. This means that authors should either prepare their source code in non-commercial software and make it publicly available or develop an application with a graphical user interface, enabling the introduction of molecular structures. Clearly, the first option would require programming skills from end-users. To the best of our knowledge, of the reports cited, only two research groups published their models in such a way that the scientific community could benefit from them.

Rivera-Delgado et al. [38] developed two separate ensemble ML models for α- and β-CD, consisting of the following methods: Cubist, GBM, MARSplines, PLS, RF, as well as SVR using polynomial, RBF, and sigmoid kernels. The algorithms were developed in the R programming language using the open-source PaDEL descriptors [55]. A publicly available application has been developed [97] to predict the Δ*G* for a compound of interest and to check whether the chemical falls within the AD of the method. Using this application requires basic programming knowledge, as the repository needs to be downloaded from the author’s website on the GitHub server and run using RStudio software. Additionally, it is necessary to install all packages required by the application.

The LightGBM model developed by Zhao et al. [43] is available on the FormulationAI website [93,98]. The user should enter the SMILES representation for the compound of interest, as well as the desired pH and temperature. Then, within a few seconds, the model determines Δ*G*s for complexes with the following CDs: native α-, β-, and γ-CD; and substituted HP-, SBE-, RM-, dimethyl-, and trimethyl-β-CD.

## 3. Other ML Applications in the Field of CD Complexes

So far, predictions of the thermodynamic stability of CD complexes have been discussed. In these models, the descriptors determined for molecules from the dataset acted as input variables (*x*), while the experimental Δ*G*, ln⁡K, and log⁡K values were treated as output variables (*y*). However, QSAR/QSPR methods are generally not limited to this particular scheme. Instead of computed molecular descriptors, spectral information or experimental conditions can also be used as the *x* values. Predictions may concern not only thermodynamic stability but also the physicochemical properties of the complex (e.g., yield, solubility, chemical stability, water content), or they may be related to a specific analytical technique (such as the concentration of the analysed substance or the resolution between two peaks in separation techniques). It is frequently observed that the ANN algorithm facilitates the construction of a good predictive model [99,100,101,102,103,104].

Unlike regression models, the purpose of classification models is only to predict whether an inclusion complex will form or not. Experimentally, the differential scanning calorimetry (DSC) technique allows for a simple assessment of whether a sample is an inclusion complex or a simple physical mixture of components. Alternatively, inclusion complexes can be distinguished from physical mixtures using infrared spectra; however, their interpretation is not straightforward [105]. To facilitate this task, Mizera et al. [92] developed an algorithm for predicting complex formation in the tested sample based solely on infrared spectra. The dataset (*n* = 42) used in model training consisted of infrared spectra as *x* variables and true/false results from DSC as *y* variables. Evaluation of the developed model showed that the predictions were correct for 90.1% of the data points.

Some authors report the use of ML algorithms to find the optimal experimental conditions for the preparation of CD complexes. In this approach, a set of experiments is performed under different conditions, then the properties of the resulting complex are examined, and finally the data are fed into an ML algorithm to find the best conditions. This procedure allows for a significant reduction in the number of experiments needed to find optimal variables. For example, the concept of design of experiment (DoE) was used to optimise a formulation consisting of doxycycline, HP-β-CD, and Mg^2+^ [99]. The main goal of these studies was to increase the chemical stability of doxycycline in aqueous solution, i.e., to inhibit the degradation process of this API. Initially, a series of 30 experiments were performed by changing four variables: the molar ratios of HP-β-CD/doxycycline (*X*_1_) and Mg^2+^/doxycycline (*X*_2_), the inclusion time (*X*_3_), and the temperature (*X*_4_). Two responses were determined for each experiment, namely the inclusion efficiency (*Y*_1_) and the stability of doxycycline (Y_2_). In order to find the optimal conditions for the preparation of the complex, the following methods were used: response surface methodology (RSM), ANN, and SVR. Among them, an ANN with one hidden layer containing ten neurons performed best in determining the set of optimal values for *X*_1_–*X*_4_. An experiment was performed using these conditions, and the resulting yield and chemical stability of doxycycline were found to be higher compared to the results of all 30 initial experiments.

A similar study aimed to optimise the spray-drying instrumental parameters to obtain a beneficial formulation containing aripiprazole and HP-β-CD [100]. In the series of performed experiments, three input variables of feed concentration, pump speed, and inlet air temperature influenced three response values of yield, moisture content, and outlet air temperature. The authors investigated the usefulness of the combined use of quality-by-design tools, such as central composite design, RSM, and ANNs. The best predictability was obtained in the case of ANNs, whereas the equations derived from RSM contributed to a better understanding of the relationships between the input and output variables.

Among the benefits of forming CD inclusion complexes, a significant advantage is the improved solubility of the guest. In the ML study by Wang et al. [106], input variables included the composition of the clathrate (drug content, molecular weight of the drug, and CD/drug ratio) and the operating conditions (drug concentration, pH, pressure, temperature, and dissolution time). The optimised RF algorithm achieved an *R*^2^ value of 0.89 when predicting the percentage of drug dissolved and 0.91 when predicting the dissolution efficiency. Among the used input variables, the molecular weight and the time of dissolution of the drug were found to be the most important.

One strategy to increase drug solubility is to obtain a ternary system consisting of the drug, CD, and polymer [10,107]. However, there is an extremely large number of possible polymers; therefore, ternary formulations are usually tested by trial-and-error approach, which is labour-intensive and wastes materials. Li et al. [96] developed predictive models for ternary CD formulations based on 596 data points taken from the literature. The RF model achieved good performance in predicting the solubility of ternary complexes and the solubility ratio between ternary and binary complexes. Based on the obtained feature importance ranking, great significance was assigned to the low melting point and hydrophobicity of the drug molecule but not to the size match with the CD cavity. Therefore, the authors hypothesised that, unlike binary systems, in ternary systems the drug may not enter the CD cavity.

In analytical chemistry, one of the advantages of using CDs is the significant enhancement of the fluorescence of the guest included in the CD cavity. Ng and Narayanaswamy [101] developed a spectrofluorimetric method for the determination of *N*-phenyl-1-naphtylamine (NPN), a widely used but toxic compound. In the presence of β-CD, significant NPN fluorescence was observed, accompanied by a shift in the maximum emission wavelength. In order to broaden the range of NPN concentrations that can be determined using this method, an ANN was developed with the input neurons corresponding to fluorescence intensities measured at specified wavelengths. Each data point in the dataset corresponded to a different NPN concentration. The results obtained using the optimised ANN extended the analytical range of the method by 71%.

Another analytical application of CDs involves the separation of enantiomers by capillary electrophoresis. Using this technique, Asensi-Bernardi et al. [108] conducted a QSPR study consisting of two stages. First, a PLS-based method was developed to predict the separation of enantiomers from racemic mixtures using highly sulphated β-CD as a chiral selector added to the background electrolyte (BGE). In this part of the study, the structural features of 40 tested basic compounds were used. Among them, the most important were: (i) the octanol–water partition coefficient determined at a pH of 7.4; (ii) the polar surface area; (iii) the number of hydrogen bond donors; and (iv) the number of hydrogen bond acceptors. Following this, the Box–Behnken experimental design equipped with the PLS algorithm was used to find the optimal experimental conditions for the separation of bupivacaine enantiomers. Based on the results of 15 experiments, the resolution between peaks of enantiomers was expressed by a linear combination of three variables: CD concentration, temperature, and pH of the BGE.

Another study used the HPLC technique, where β-CD was covalently bonded to the chromatographic column filling, thus creating a new stationary phase [109]. It was found that for the set of 31 indole derivatives, the stability of the inclusion complexes formed between the analyte and the stationary phase determined the retention times of the analytes. To predict the retention factors of the studied compounds, a QSPR model was developed using the stepwise MLR method, with the experimentally derived retention factors as *y* values and the computed molecular descriptors as *x* values. The results showed that the binding of the tested compounds to β-CD is strongly affected by the length and flexibility of the side chain, which is attached to the indole scaffold and contains the carboxyl group.

In reversed-phase HPLC separations, increasing the content of environmentally unfriendly acetonitrile in the mobile phase generally reduces the retention times of hydrophobic substances. It has been pointed out that the addition of CD to the mobile phase also results in decreased retention times, but the underlying mechanism is complicated and involves complexation and adsorption equilibria [102,103,104]. To enable the optimal separation of seven compounds, including risperidone and olanzapine, quantitative structure–retention relationship (QSRR) studies were performed. The ANN input variables included both the molecular descriptors of the studied compounds, the chromatographic conditions used, and the energetic properties of the inclusion complex determined in the docking study. The HPLC method with optimal parameters identified in the QSRR study was validated in terms of linearity, accuracy, and precision. In addition, the tests of ‘greenness’ (eco-friendliness) achieved 90 out of 100 possible points.

Natural language processing (NLP) is a significantly different branch of ML. Unlike all previously discussed models, the NLP algorithms operate with words, not numbers. The goal of NLP is to enable computers to ‘understand’ the content of the text documents written with human speech. In the case of CD complexes, this technique was used to automate the processing of 1998 patents to make statistical summaries without the need for humans to read the full documents [110,111]. The developed algorithm consisted of the following steps: (i) pre-processing of the text, involving the removal of punctuation marks and deleting common words unrelated to CD applications, etc.; (ii) specifying the most frequently occurring words in all of the patents; (iii) describing each patent with a vector containing the frequencies of finding these words; (iv) calculating the similarity factor for all the pairs of patents; (v) generating a network in which a link between the patents exists when their similarity factor is high enough; and (vi) cluster analysis of this network [110,111]. Among the many summaries obtained in this way, it is worth mentioning the graphs that illustrate the time-dependence of the number of patents on CD complexes, grouped according to a predefined criterion. The use of a text-mining algorithm allowed for the division of patents according to the purpose of CD complex formation (increasing solubility, stability, and taste masking), route of administration (parenteral, oral, ocular, etc.), and dosage form (solution, powder, tablet, etc.) [110,111].

## 4. Summary and Concluding Remarks

This article reviews the QSAR/QSPR models developed for making predictions for CD complexes. Most of the models discussed concerned the thermodynamic stability of the complexes. The constituents of the methods, i.e., datasets, descriptors, and ML algorithms, were discussed in detail, showing the progress achieved over time and indicating some directions of development for the future.

Over the last two decades, a significant change has been observed in the size and content of datasets. Previously, models often used small datasets containing chemically similar compounds, such as alkanols, barbituric acid derivatives, or substituted benzenes. In this review, we showed that the authors used datasets from various sources, containing 60–3000 compounds with various structures. Consequently, current methods possess a considerably wider AD, but due to the use of different datasets, it is difficult to compare their predictive capabilities with each other, and it is practically impossible to select one model that performs best. Due to the ever-increasing number of articles reporting experimental results on the thermodynamic stability of CD complexes with various guests, we hope that the size of the available datasets will increase over time. However, caution should be taken to distinguish results obtained for complexes with randomly substituted CDs according to the claimed DS of the CD used, as this property may affect complex formation. There is also a need to gather results obtained at different pH values, which will enable the construction of a model describing a wide range of dependence of thermodynamic stability on the pH value.

The discussed works use many different approaches to generate descriptors. The early QSAR/QSPR models took advantage of a relatively small number of descriptors, mainly limited to topological ones. Thanks to the considerably improved access to computational power in the last decade, it is now feasible to find the optimal 3D structures for all the compounds from a dataset, thus allowing for the determination of their 3D descriptors. Due to the availability of various software from different vendors, several thousands of descriptors may be calculated for each molecule, with the most important descriptors then selected using specialised ML techniques. However, it was observed that the lists of the most significant descriptors are not the same between different works, and their meaning is often neither straightforward nor intuitive. In the papers discussed, most authors developed single models for each CD, which means that they calculated descriptors only for guest molecules. In a recent approach, single models included a larger number of CDs (3–16), and descriptors were computed for both the host and the guest. However, the procedure for calculating descriptors for randomly substituted CDs was not clearly explained in that case. It is also worth noting that only one work focused on predicting different Δ*G*s of complex formation for molecules that are a pair of enantiomers. In the future, it should be confirmed whether the selected set of descriptors is different for compounds that are a pair of enantiomers and, consequently, whether this difference is reflected in the prediction of different Δ*G*s for them, as is indicated by experimental data and MD simulations.

It is also worth adding that comparing the models is even more difficult due to the use of different evaluation metrics. Depending on the measure of thermodynamic stability used (Δ*G*, ln⁡K, or $\log_{10}K$), the *MAE* and *RMSE* values are expressed differently. Therefore, they need to be converted to enable direct comparisons between different models. It is also difficult to find a report that presents all the following evaluation metrics: *R*^2^_train_, *R*^2^_test_, *Q*^2^_LOO_, *MAE*_test_, and *RMSE_t_*_est_. Although QSAR methods generally aim to obtain predictions for new compounds, only two of the models discussed here have been made available to the scientific community. One of them is available as a website, while the other is a graphical application that requires the installation of the RStudio program.

This work compares the capabilities of the QSAR/SQPR method with those offered by molecular modelling. Since the latter allows for the prediction of the 3D shape of an inclusion complex, it can be considered superior in terms of interpretability. On the other hand, the QSAR/QSPR approach may be beneficial for predicting the stability of complexes of randomly substituted CDs without detailed knowledge of their chemical composition. ML techniques should also allow for the prediction of the pH-dependence of Δ*G* without the necessity of determining pK_a_ values of ionisable functional groups and specifying dominant protonation forms. Moreover, QSAR/QSPR methods do not make a simplifying assumption about neglecting the aggregation of species in solutions of CD complexes and their constituents.

It is worth noting that QSAR/QSPR studies are not limited to predictions of the thermodynamic stability of complexes. Scientists have managed to predict the physicochemical properties of complexes, such as the solubility, an increase in fluorescence, changes in migration time in capillary electrophoresis, and retention time in HPLC. The ML approach was also used to find the optimal experimental conditions for the formation of a CD complex.

## Figures and Tables

**Table 1:** Metrics for evaluating regression models.

$R^{2}=\frac{\Sigma_{i}{\left({\hat{y}}_{i}-\bar{y}\right)}^{2}}{\Sigma_{i}{\left(y_{i}-\bar{y}\right)}^{2}}=1-\frac{\Sigma_{i}{\left(y_{i}-{\hat{y}}_{i}\right)}^{2}}{\Sigma_{i}{\left(y_{i}-\bar{y}\right)}^{2}}$
$Q^{2}=1-\frac{\Sigma_{i}{\left(y_{i}-{\hat{y}}_{i}\right)}^{2}}{\Sigma_{i}{\left(y_{i}-\bar{y}\right)}^{2}}$
$mean\;absolute\;error\;(MAE)=\frac{1}{n}\sum_{i=1}^{n}\left|{\hat{y}}_{i}-y_{i}\right|$
$root\;mean\;square\;error\;(RMSE)=\sqrt{\frac{1}{n}\sum_{i=1}^{n}{\left({\hat{y}}_{i}-y_{i}\right)}^{2}}$
$y_{i}$ = experimental value for the *i*-th data point
${\hat{y}}_{i}$ = predicted value for the *i*-th data point
$\bar{y}$ = mean of all experimental values in the dataset

**Table 2:** Summary of predictions for Δ*G*, ln⁡K, and log⁡K of complex formation with α-CD, arranged in chronological order.

*y*	n_dataset_ [Source]	n_training_/n_test_, Split Algorithm	Descriptors	Algorithms, Best Algorithm (Software)	Performance	AD Check	Ref.
ln⁡K	72 [89]	54/18, largest minimum distance	VolSurf, CoMFA	SVR, PLS (MATLAB, PLS_toolbox)	R^2^_train_ = 0.84, R^2^_test_ = 0.89, Q^2^_LOO_ = 0.68	-	[69]
log⁡K	60 [90]	45/15, hierarchic bin system	standard CoMFA, COSMOsar3D	PLS	R^2^_test_ = 0.70, Q^2^_leave-two-out_ = 0.83, RMSE_test_ = 2.57 kJ/mol	-	[67]
log⁡K	195 (from 17 sources)	146/49, random division	DRAGON	stepwise MLR (SPSS software)	R^2^_test_ = 0.77, Q^2^_LOO_ = 0.84, MAE_test_ = 2.17 kJ/mol, RMSE_test_ = 2.82 kJ/mol	+	[74]
Δ*G*	about 200 [40,44]	75%/25%, R package caret	PaDEL	ensemble of: Cubist, GBM, MARSplines, RF, polynomial SVR, RBF SVR (Rstudio)	for single models: R^2^_test_ from 0.63 to 0.78, Q^2^_LOO_ from 0.50 to 0.64	+	[38]
log⁡K	186 [40,44,91]	149/37	PaDEL or molecular fingerprints	ensemble of: RBF regression, GPR, RF, GBT, tree ensemble (KNIME)	R^2^_test_ = 0.84, MAE_test_ = 1.37 kJ/mol RMSE_test_ = 1.71 kJ/mol	+	[58]
log⁡K	229	183/46	norm indices	MLR	R^2^_train_ = 0.92, R^2^_test_ = 0.93, Q^2^_LOO_ = 0.89, MAE_test_ = 1.03 kJ/mol	-	[61]

The following abbreviations are used: AD—applicability domain; CoMFA—comparative molecular field analysis; GBM—gradient-boosting machine; GBT—gradient-boosted trees; GPR—Gaussian process regression; MARSplines—multivariate adaptive regression splines; MLR—multiple linear regression; n_dataset_, n_training_, n_test_—the number of data points in the entire dataset, training set, and test set, respectively; PLS—partial least squares; RBF—radial basis function; RF—random forest; SVR—support vector regression; *y*—the quantity which is predicted.

**Table 3:** Summary of predictions for Δ*G*, ln⁡K, and log⁡K of complex formation with β-CD, arranged in chronological order.

*y*	n_dataset_ [Source]	n_training_/n_test_, Split Algorithm	Descriptors	Algorithms, Best Algorithm (Software)	Performance	AD Check	Ref.
log⁡K	233 [40]	173/60, most descriptive compound	GRIND (Pentacle)	PLS (Pentacle)	R^2^_train_ = 0.87, R^2^_test_ = 0.74 Q^2^_LOO_ = 0.75	+	[68]
Δ*G*	218 [41]	196/22, maximum dissimilarity algorithm	MOE 2D, Moriguchi and Blake, SMARTS keys, Erlangen 2D	Cubist, RF	R^2^_train_ = 0.996, R^2^_test_ = 0.95 MAE_test_ = 1.54 kJ/mol, RMSE_test_ = 2.09 kJ/mol	-	[78]
log⁡K	233 [42]	186/47, k-means cluster analysis	derived from electrostatic potentials on the molecular surface	MLR, PLS, SVR, least squares SVR, RF, GPR (ZP-explore in MATLAB)	R^2^_train_ = 0.93, R^2^_test_ = 0.83 Q^2^_LOO_ = 0.76, RMSE_test_ = 2.13 kJ/mol	-	[62]
log⁡K	126 [36]	98/28, Kennard–Stone algorithm	GRIND (Pentacle)	PLS (MATLAB, PLS_toolbox)	R^2^_train_ = 0.76, R^2^_test_ = 0.68 Q^2^_LOO_ = 0.64	-	[37]
log⁡K	233 [42]	186/47, k-means cluster analysis	norm indexes	PLS, ANN, MLR, least squares SVR	R^2^_train_ = 0.91, R^2^_test_ = 0.83 Q^2^_LOO_ = 0.77	-	[60]
Δ*G*	218 [41]	160/58, DUPLEX	DRAGON	stepwise MLR, ANN	R^2^_train_ = 0.94, R^2^_test_ = 0.96, MAE_test_ = 0.93 kJ/mol	+	[73]
log⁡K	233 [42]	70 (sub-training)/69 (calibration)/47 (validation)/47 (test)	molecular optimal descriptor DCW	Monte Carlo + linear regression (CORAL)	R^2^_test_ = 0.93, MAE_test_ = 1.10 kJ/mol	+	[59]
log⁡K	233 [40]	170/63, random division	for ligand and complex (MOE, AutoDockTools, BINANA)	MLR	R^2^_train_ = 0.83, R^2^_test_ = 0.78 Q^2^ = 0.82	+	[75]
Δ*G*	76 [44]	n.a.	3D fragment molecular descriptors (mfSpace)	singular value decomposition for MLR (TRAIL, ISIDA QSPR)	R^2^ = 0.92 RMSE = 1.1 kJ/mol	+	[65]
log⁡K	324 [37,40]	243/81, random split	spectrophores	RF (scikit-learn)	R^2^_train_ = 0.95, R^2^_test_ = 0.66, MAE_test_ = 2.28 kJ/mol, RMSE_test_ = 2.91 kJ/mol	-	[63]
ln⁡K	233 [40]	187/46, activity division approach	Chemopy, CDK, RDKit, Pybel, BlueDesc, PaDEL (ChemDes, BioCCI)	MARSplines (STATISTICA 12)	R^2^_train_ = 0.91, R^2^_test_ = 0.94, Q^2^_LOO_ = 0.90, MAE_test_ = 1.09 kJ/mol	-	[70]
Δ*G*	about 250 [36,40]	75%/25%, R package caret	PaDEL	ensemble of: Cubist, GBM, MARSplines, RF, polynomial SVR, RBF SVR (Rstudio)	for single models: R^2^_test_ from 0.73 to 0.85 Q^2^_LOO_ from 0.56 to 0.73	+	[38]
log⁡K	232 [40]	186/46	PaDEL or molecular fingerprints	ensemble of: RBF regression, GPR, RF, GBT, tree ensemble (KNIME)	R^2^_test_ = 0.89 MAE_test_ = 1.26 kJ/mol RMSE_test_ = 1.61 kJ/mol	+	[58]
true/false	200 (own)	140/60, random division	MOE	ensembles of DNN, SVM, LR (scikit-learn, numpy)	depending on the model	-	[76]
log⁡K	330	264/66	norm indices	MLR	R^2^_train_ = 0.91, R^2^_test_ = 0.92 Q^2^_LOO_ = 0.90, MAE_test_ = 1.09 kJ/mol	-	[61]

An explanation of the selected abbreviations can be found under Table 2 and below: ANN—artificial neural network, DNN—deep neural network; LR—logistic regression; SVM—support vector machine.

**Table 4:** Summary of predictions of complex formations not included in Table 2 and Table 3, arranged in chronological order.

Host	*y*	n_dataset_ [Source]	n_training_/n_test_, Split Algorithm	Descriptors	Algorithms, Best Algorithm (Software)	Performance	AD Check	Ref.
SBE-β-CD	Δ*G*	220	198/22, maximum dissimilarity algorithm	MOE 2D, Moriguchi and Blake, SMARTS keys, Erlangen 2D	Cubist, RF	R^2^_train_ = 0.94, R^2^_test_ = 0.92, MAE_test_ = 2.12 kJ/mol, RMSE_test_ = 2.71 kJ/mol	-	[78]
7 CDs	true/ false	42 (own)	n.a.	infrared spectra	ExtraTreesClassifier (TPOT, scikit-learn)	accuracy 90.1%	-	[92]
8 CDs	Δ*G*	3000	80% (training)/ 10% (validation)/ 10% (test)	17 for guests, 22 for CDs + pH + T (ALOGPS, ChemAxon)	RF, DNN, LightGBM (scikit-learn, Keras)	R^2^_train_ = 0.86, R^2^_test_ = 0.86, MAE_test_ = 1.38 kJ/mol, RMSE_test_ = 1.83 kJ/mol	-	[43,93]
16 CDs	true/ false	1654	80%/20%, random division	PaDEL (Modred)	GB of DTs (LightGBM)	depending on the threshold	-	[45]
3 CDs	Δ*G*	725 [36]	95%/5%	PaDEL for guests and CDs	DNN	R^2^_train_ = 0.98, R^2^_test_ = 0.996, MAE_test_ = 0.771 kJ/mol	-	[39]
8 CDs	Δ*G*	2992 [43]	2318 (test)/339 (validation)/335 (test), stratified random sampling	19 for guests, 21 for CDs + pH + T (ALOGPS, ChemAxon)	SVR, DNN, LightGBM, AttPharm (scikit-learn, Keras, TensorFlow)	R^2^_train_ = 0.88, R^2^_test_ = 0.86, MAE_test_ = 1.34 kJ/mol	-	[81]
3 CDs	Δ*G*	280 [94]	224/56, stratified k-fold method	9 for guests, 10 for CDs + pH + T (RDKit, KNIME)	SVR	R^2^_train_ = 0.92, R^2^_test_ = 0.78, MAE_test_ = 1.35 kJ/mol, RMSE_test_ = 1.93 kJ/mol	-	[77]
3 CDs	Δ*G*	280 [94]	224/56, stratified k-fold method	9 for guests, 10 for CDs + pH + T (RDKit, KNIME)	SVR, XGB, GPR	R^2^_train_ = 0.95, R^2^_test_ = 0.80, MAE_test_ = 1.20 kJ/mol, RMSE_test_ = 1.81 kJ/mol	+	[53]

An explanation of the selected abbreviations can be found under Table 2 and below: DT—decision tree; GB—gradient boosting; XGB—extreme gradient boosting.

**Table 5:** Confusion matrix and evaluation measures for classification models.

		Experimental	
		complex formed	complex not formed
**Predicted**	**complex formed**	$n_{TP}$	$n_{FP}$
	**complex not formed**	$n_{FN}$	$n_{TN}$
$accuracy=\frac{n_{TP}+n_{TN}}{n_{TP}+n_{FN}+n_{FP}+n_{TN}}$ $precision=\frac{n_{TP}}{n_{TP}+n_{FP}}$ $recall=\frac{n_{TP}}{n_{TP}+n_{FN}}$			
